# Editor’s Cut: The First 10 Years of *JACC: Basic to Translational Science*

**DOI:** 10.1016/j.jacbts.2025.04.002

**Published:** 2025-05-26

**Authors:** Douglas L. Mann

**Figure:**
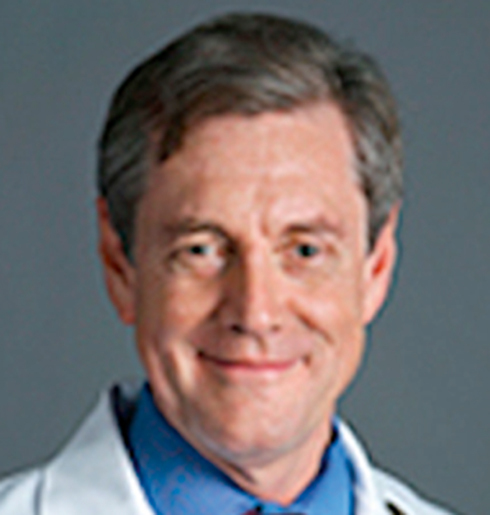

One of the privileges and honors that comes with being an Editor-in-Chief of a *JACC* Journal is the opportunity to write an Editor’s Page for each issue. Over the years, the editorials I have written have spanned a broad range of different topics both within and beyond cardiovascular medicine, impacted by current events, topics of pressing interest (COVID, COVID, COVID), concepts I thought were important to the field of translational science, and—on rare occasion—fun stuff that I wanted to share with colleagues.

The snarky comment that “good artists copy, great artists steal” has been attributed (without clear documentation) to the artist Pablo Picasso. For this, my penultimate Editor’s Page as Editor-in-Chief of *JACC: Basic to Translational Science*, I wanted to “borrow” an idea from my good friend and the former Editor-in-Chief for *JACC: Heart Failure* (2013-2022), Dr Christopher O’Connor, who focused one of his final Editor’s Pages on his top 10 Editor’s Pages he felt were notable both in terms of the number of citations and the preference of the readership.^1^ Here, I wanted to take a slightly different tack by highlighting select editorials from each of the past 10 years that reflect how *JACC: Basic to Translational Science* has sought to frame and interpret the changing landscape of medicine and science, sculpted by the broader social, political, and cultural realities of our time.

## 2016

Shah AM, Mann DL. Potential effect of Brexit on cardiovascular translational science. *JACC Basic Transl Sci.* 2016;1:416-417.

## 2017

Mann DL. Deus ex machina: why mechanism matters in translational research. *JACC Basic Transl Sci.* 2017;2:227-228.

## 2018

Mann DL. Fake news, alternative facts, and things that just are not true: can science survive the post-truth era? *JACC Basic Transl Sci.* 2018;3:573-574.

## 2019

Schafer AI, Mann DL. Current education of physicians: lost in translation? *JACC Basic Transl Sci.* 2019;4:655-657.

## 2020

Mann DL. Chinese health care workers and COVID-19: for whom the bell tolls. *JACC Basic Transl Sci.* 2020;5:415-417.

## 2021

Mann DL. Now that we have an effective vaccine for COVID-19, will it also inoculate us against the virus of indifference? *JACC Basic Transl Sci.* 2021;6:86-87.

## 2022

Mann DL. Fake it till you make it: what every translational investigator can learn from the rise and fall of Theranos. *JACC Basic Transl Sci.* 2022;7:99-100.

## 2023

Mann DL, Rasmussen LM. Who should be accountable for scientific accountability? *JACC Basic Transl Sci.* 2023;8:1040-1042.

## 2024

Mann DL. Should all translational scientists be Swifties? *JACC Basic Transl Sci.* 2024;9:440-442.

## 2025

Mann DL. When facts become forbidden: the past and present history of scientific censorship. *JACC Basic Transl Sci.* 2025;10:402-404.

I have never thought that editorials should be viewed as the definitive word on a topic. Rather, an editorial should serve as a platform to stimulate dialogue and promote further discussion. To this end, I have been appreciative of the many comments from colleagues who have reached out, not only to agree, but also at times to offer differing perspectives on the themes covered in the editorial pages of *JACC: Basic to Translational Science*. Please know that I am grateful for your thoughts and thank you for your support of the journal over the past 10 years.
